# Probiotics improve symptoms of patients with COVID-19 through gut-lung axis: a systematic review and meta-analysis

**DOI:** 10.3389/fnut.2023.1179432

**Published:** 2023-05-22

**Authors:** Yong Tian, Hongmei Ran, Xudong Wen, Guochuan Fu, Xiaofang Zhou, Rui Liu, Tao Pan

**Affiliations:** ^1^Clinical Medical College, Chengdu University of Traditional Chinese Medicine, Chengdu, Sichuan, China; ^2^Department of Gastroenterology, Chengdu Integrated TCM and Western Medicine Hospital, Chengdu, Sichuan, China

**Keywords:** nutrition – clinical, probiotics, gut–lung axis, COVID−19, meta–analysis

## Abstract

**Background:**

Multi system symptoms such as gastrointestinal tract and respiratory tract exist in coronavirus disease 2019 (COVID-19) patients. There is a lack of reliable evidence to prove that probiotics are effective in improving these symptoms. In this study, we aimed to evaluate the efficacy of probiotics in meta-analysis.

**Methods:**

We systematically searched PubMed, Embase, Web of Science, and Cochrane Library up to February 15, 2023. Randomized controlled trials or high quality retrospective studies comparing the efficacy of probiotics as supplementation with non-probiotics in improving symptoms for patients with COVID-19 were included. This meta-analysis assessed endpoints using Review Manager 5.3.

**Result:**

Ten citations comprising 1198 patients with COVID-19 were included. The results showed that probiotics could increase the number of people with overall symptom improvement (RR = 1.62, 95% CI [1.10, 2.38], *P* = 0.01) and shorten the duration (days) of overall symptoms (MD = −1.26, 95% CI [−2.36, −0.16], *P* = 0.02). For the duration (days) of specific symptoms, probiotics could improve diarrhea (MD = −2.12, 95% CI [−2.41, −1.83], *P* < 0.00001), cough (MD = −2.21, 95% CI [-4.56, 0.13], *P* = 0.06) and shortness of breath (MD = −1.37, 95% CI [-2.22, −0.53], P = 0.001). Probiotics had no obvious effect on fever, headache and weakness. For inflammation, probiotics could effectively reduce C-reactive Protein (CRP) serum level (mg/L) (MD = −4.03, 95% CI [−5.12, −2.93], *P* < 0.00001). Regarding hospital stay (days), probiotics group was shorter than non-probiotics group (MD = −0.98, 95% CI [−1.95, −0.01], *P* = 0.05).

**Conclusion:**

To some extent probiotics could improve the overall symptoms, inflammatory reaction and shorten hospital stay of patients with COVID-19. Probiotics may improve gastrointestinal symptoms (such as improving intestinal flora and reducing the duration of diarrhea) and further improve respiratory symptoms through the gut-lung axis.

**Systematic review registration:**

https://www.crd.york.ac.uk/PROSPERO/display_record.php?RecordID=398309, identifier: CRD42023398309.

## Introduction

Since the severe acute respiratory syndrome coronavirus 2 (SARS – CoV2) pandemic in 2019, the pathogen has undergone a variety of mutations, including Delta and Omicron. The symptoms after infection vary from mild to severe, not only in the respiratory system, but also in the digestive and nervous systems. Although fever, headache and cough are the most common clinical features of COVID-19, symptoms of gastrointestinal involvement (such as diarrhea, nausea and vomiting) are increasing (1, 2). In fact, there is a close relationship between lung and gut. The bidirectional interactions between the respiratory mucosa and the gut microbiota, known as gut-lung axis, are supposed to be involved in the healthy or pathologic immune responses to SARS-CoV-2 (3). Through the gut-lung axis, gut dysbiosis may also affect the pathogenesis of the lung and change the clinical outcomes in COVID-19 (4). Therefore, the application of nutraceutical agents to improve the composition and diversity of intestinal flora may have a positive impact on the prevention/treatment of COVID-19.

Probiotics is a kind of active microorganism beneficial to the host that changes the composition of a certain part of the host flora by colonization in the human body (5). At present, probiotics, including *Bifidobacteria, Saccharomyces boulardii* and *Lactobacillus*, are mainly used in clinical supplementation to improve intestinal flora (6–8). As well known, probiotics can reinforce immunity and counteract inflammation by restoring symbiosis within the intestinal microbiota. They have a potential role in the treatment of viral respiratory infections and influenza virus infections, as well as in the treatment of nervous system diseases (9). These effects may stem from the regulation of several potential mechanisms (10–16), including cell phenotype, endocrine factors, and signaling pathways (Table 1). For example, probiotics improve asthma symptoms and lung inflammation maybe through increasing Treg cells population and regulating TLR4/NF-kB signaling pathways, respectively. For influenza viruses, probiotics can activate human and cellular immune responses to increase IFN- γ and IL-2, thereby increasing the host's resistance to influenza virus infection. Many researchers believe that probiotics may have a potential effects on patients with COVID-19 (4, 17, 18). However, there is still a lack of reliable research evidence on the impact of probiotics for COVID-19 patients. Therefore, we performed a meta-analysis to assess the efficacy of probiotics in improving the symptoms of patients with COVID-19.

**Table 1:** Potential mechanism of probiotics in the treatment of respiratory and neurological diseases.

**Study**	**Disease**	**Mechanisms**
Abbasi-Dokht et al. (10)	Asthma	Multistrain probiotics supplement alleviates asthma symptoms *via* increasing Treg cells population.
Wu et al. (11)	Allergic asthma	Probiotics and prebiotics may treat allergic asthma inflammation and pneumonia induced by OVA-LPS by regulating TLR4/NF-kB signaling pathways.
Karim et al. (12)	Chronic obstructive pulmonary disease	Multistrain probiotic improves muscle strength and functional performance in chronic obstructive pulmonary disease patients by reducing intestinal permeability and stabilizing neuromuscular junction.
Kokubo et al. (13)	Cold	*Lactococcus lactis* may improve cold-like symptoms and fatigue feelings by stimulating plasmacytoid dendritic cells.
Song et al. (14)	Influenza virus	*Lactobacillus rhamnosus* M21 can activate humoral and cellular immune responses to increase IFN- γ And IL-2, thereby increasing the host's resistance to influenza virus infection.
Tan et al. (15)	Traumatic brain injury	Daily prophylactic administration of probiotics could attenuate the deviated Th1/Th2 response induced by severe traumatic brain injury, and could result in a decreased nosocomial infection rate.
Piletz et al. (16)	Neurological disorders	*Lactobacillus rhamnosus* or *Lactobacillus fermentans* stimulate neurite growth of SH-SY5Y through the gut-brain axis, thereby altering brain function, behavior, and mental and neurological disorders.

## Materials and methods

### Retrieval strategy

We performed a comprehensive online search of PubMed, Embase, Web of Science, and Cochrane Library from database establishment to February 15, 2023. The following search terms were included: (“probiotics” OR “synbiotics” OR “prebiotics” OR “postbiotics”) AND (“COVID-19” OR “2019 nCoV Disease” OR “coronavirus disease 2019” OR “novel coronavirus” OR “SARS-CoV-2”). Relevant publications that compared probiotics as supplementation with non-probiotics (including placebo, standard care and no probiotics received) for improving the clinical symptoms of patients with COVID-19 would be taken into consideration. Symptoms, inflammation, and hospital stay were our main indicators for evaluating the efficacy of probiotics. No language or national restrictions were imposed. To ensure high quality of the work, we performed the systematic review and meta-analysis according to the Preferred Reporting Items for Systematic Reviews and Meta-Analyses (PRISMA) statement (19). We registered our research on the International Prospective Register of Systematic Reviews (PROSPERO), and the registration number was CRD42023398309.

### Inclusion and exclusion criteria

Potentially relevant published studies underwent a review of the entire published manuscript. The selection criteria for inclusion in the meta-analysis: (1) Randomized controlled clinical trials (RCTs) were the main types of studies included, and high quality retrospective studies (RETRO) would also be considered when it could provide reliable data for outcome indicators; (2) Study subjects were patients with COVID-19, with no restrictions considering sex, age, race, and disease duration; (3) Studies evaluating the efficacy of probiotics as supplementation vs. non-probiotics in improving the symptoms of patients with COVID-19 were included. The exclusion criteria were as follows: (1) Crossover studies or single-arm studies that do not meet the inclusion criteria will be excluded; (2) Studies without probiotics as intervention measures or without reasonable control measures will be excluded; (3) Duplicate publications, review articles, editorials, case reports, and animal experiments were excluded. The decision to include or exclude the published studies was made separately by two researchers. Any disagreement would be resolved through discussion until a consensus is reached by consulting the third author.

### Data extraction and quality assessment

Through detailed reading of the final included studies, two reviewers extracted the required data using standardized formats. The following data needed to be included: first author, year of publication, country, sample size, curative duration, types and dosage of intervention, number of people with overall symptom improvement, duration of symptoms (including overall symptoms, diarrhea, cough, shortness of breath, fever, cough and weakness), C-reactive protein (CRP) and hospital stay. In the original articles, most of results are presented as median [interquartile range], especially for duration of symptoms, CRP and hospital stay. Therefore, we use some equations (20, 21) to convert them into available data of mean ± standard deviation (Supplementary material S1). Herein, overall symptoms, CRP and hospital stay were examined as the main outcomes. Specific symptoms such as diarrhea, cough, shortness of breath, fever and headache were the additional outcomes. We used the Newcastle–Ottawa scale (NOS) as the tool to estimate the quality of the retrospective studies. Studies with a score of 7 or higher were considered to be high quality. Jadad scale were used for quality assessment and determining the risk of bias in identified RCTs, and scores of 4 or higher were regarded as high quality.

### Data analysis

The included studies were tested for heterogeneity using Cochran's *Q*-test and I^2^ test (when *P* < 0.10 was considered significant). According to the Cochrane handbook, the fixed-effects model was selected for no obvious or low heterogeneity (I^2^ < 50%), and the random-effects model was selected for moderate heterogeneity (75% ≥ I^2^≥50%). When the heterogeneity among studies appeared to be high (I^2^ >75%), sensitivity analysis was performed by excluding each of the individual studies. All *P*-values were two-tailed, and a *P* < 0.05 was considered statistically significant. We estimated risk ratios (RRs) or mean difference (MD) with 95% confidence intervals (CI) were calculated to analyze variables. Outcomes were graphically represented and assessed using Review Manager 5.3. Furthermore, we selected diarrhea and hospital stay to examine publication bias using Egger's test of Stata 14.0.

## Results

### Literature search and screening

**Figure 1:**
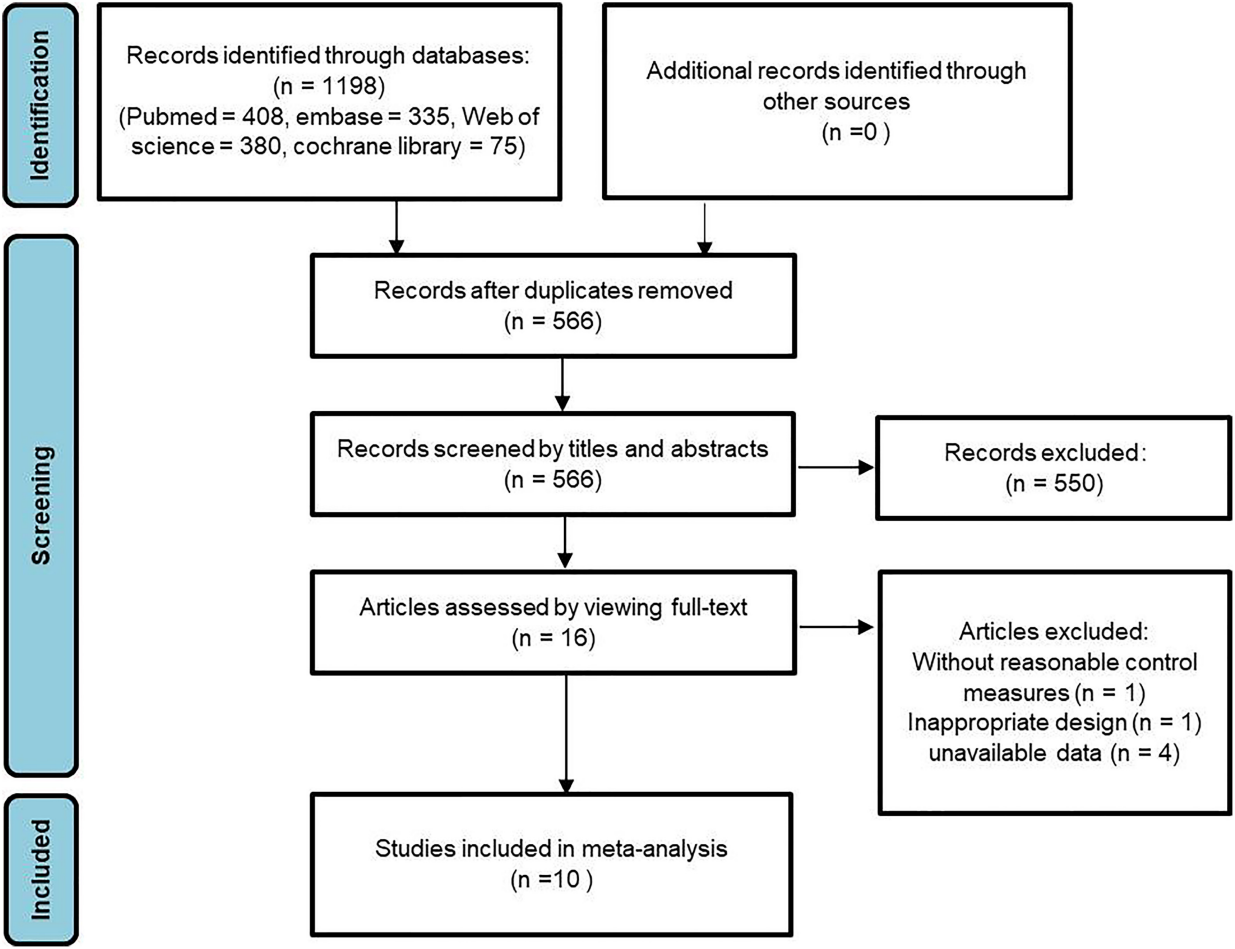
PRISMA flow diagram for study design and literature search.

In total, 1,198 studies were retrieved. Subsequently, 632 studies were excluded as duplicate publications. After reviewing the titles and abstracts, 550 articles were excluded, including reviews, editorials, and case reports. After thoroughly reading the full text of the remaining 16 articles, 2 articles with inappropriate design or without reasonable control measures were subsequently excluded. In addition, 4 articles with unavailable data were excluded. Finally, ten qualified published studies (22–31) were included in the meta-analysis. Figure 1 illustrates the study selection process.

### Literature characteristics and quality assessment

Ten studies (22–31) comprising 1,453 patients with COVID-19 were included in the analysis. They were carried out in 7 different countries, namely, Russia, Mexico, China, Spain, United States, Rome and Iran. Among these studies, 2 of them (24, 25) were retrospectively performed, and the other 8 articles (22, 23, 26–31) were RCTs. 9 studies (22–29, 31) were high quality articles, while only one (30) was of low quality. All of them were published as full-text manuscripts mainly from 2021 to 2023. There was no significant difference between the baseline data of the intervention group and the control group. Various types of probiotics were used in the intervention group, including *Lacticasseibacillus rhamnosus, Lactiplantacillus plantarum, Lactobacillus salivarius, Lactobacillus acidophilus, Pediococcus acidilactici, Bifdobacterium bifdum*, Live *Bifidobacterium longum, Bifdobacterium longum* subsp, *Pediococcus acidilactici, Streptococcus thermophilus*. Some of them have been made into tablets or capsules with different brands. We extracted and summarized the basic data of the included articles and performed quality assessment (Table 2).

**Table 2:** Characteristics of literatures and quality assessment.

**Study**	**Country**	**Study design**	**Incldued patients**	**Mean/ median ages**	**Regimens**	**Patients of group**	**Follow-up time**	**Jadad/ NOS score**
Ivashkin et al. (22)	Russia	RCT	200	T: 65 (59–71)	*Lacticaseibacillus rhamnosus* PDV 1705, *Bifdobacterium bifdum* PDV 0903, *Bifdobacterium longum* subsp. infantis PDV 1911, and *Bifdobacterium longum* subsp. *longum* PDV 2301, 4 × 10^9^ CFU Tid	99	14 days	7
				C: 64 (54–70)	No probiotics received	101	14 days	
Gutiérrez-Castrellón et al. (23)	Mexico	RCT	300	T: 34 (26–45)	*Lactiplantibacillus plantarum* KABP022, KABP023, KAPB033, and *Pediococcus acidilactici* KABP021, 2 × 10^9^ CFU Qd	150	30 days	7
				C: 39 (27–49)	Placebo	150	30 days	
Wang et al. (24)	China	RETRO	58	T: 56·32	Live *Bifidobacterium longum* > 2 × 10^7^ CFU, live *Lactobacillus bulgaricus* and *Streptococcus thermophilus* >2 × 10^6^ CFU Tid added	23	7 days	7
				C: 56.32	Standard care	35	7 days	
Zhang et al. (25)	China	RETRO	300	T: 49 (35–60)	*Bifidobacterium, Lactobacillus, Enterococcus*, 9 × 10^7^CFU Bid added	150	30 days	8
				C: 50 (37–62)	Standard care	150	30 days	
Navarro-Lopez et al. (26)	Spain	RCT	41	T: 48.88 ± 12.35	*Lactobacillus rhamnosus* CECT 30579 1 × 10^9^ CFU and *Kluyveromyces marxianus* B0399 1 × 10^8^ CFU Qd	26	30 days	5
				C: 46.33 ± 10.91	No probiotics received	15	30 days	
Wischmeyer et al. (27)	U.S.	RCT	182	T: NA	*Lactobacillus rhamnosus* GG, 2 × 10^11^ CFU Qd	91	28 days	4
				C: NA	Placebo	91	28 days	
Saviano et al. (28)	Rome	RCT	80	T: 59.2 ± 17.8	*Bifidobacterium lactis* LA 304, *Lactobacillus salivarius* LA 302, and *Lactobacillus acidophilus* LA 201, 40 × 10^9^ CFU Bid added	40	10 days	7
				C: 60.1 ± 15.2	Standard care	40	10 days	
Meskina et al. (29)	Russia	RCT	100	T: 55.5 (41–56)	*Bifidobacterium bifidum 1* and *Lactobacillus plantarum* 8P-A3, 1.5 × 10^9^ CFU Bid, 10 days; 3 × 10^8^ CFU Bid, 15 days	50	25 days	6
				C: 48.0 (41–54)	No probiotics received	50	25 days	
Maev et al. (30)	Russia	RCT	120	T: 42.27 ± 1.48	*Saccharomyces boulardii* CNCM I-745, 10 × 10^9^ CFU Qd added	60	10 days	3
				C:44.35 ± 1.40	Standard care	60	10 days	
Vaezi et al. (31)	Iran	RCT	72	T: 52.08 ± 16.08	Multi-strain probiotics, 2 × 10^9^ CFU Qd	38	14 days	6
				C: 51.54 ± 15.26	Placebo	38	14 days	

RCT, randomized controlled trial; RETRO, retrospective study; Qd, once a day; Bid, twice a day; Tid, three times a day; T, trial group; C, control group; CFU, colonyforming units; NOS, Newcastle–Ottawa scale; NA, no availability. U.S.:United States. Multi-strain probiotics: including Lactobacillus (L.) rhamnosus, L. helveticus, L. casei, Bifidobacterium (B.) lactis, L. acidophilus, B. breve, L. bulgaricus, B. longum, L. plantarum, B. bifidum, L. gasseri, and Streptococcus (S.) thermophilus.

### Meta-analysis of people with overall symptom improvement

**Figure 2:**
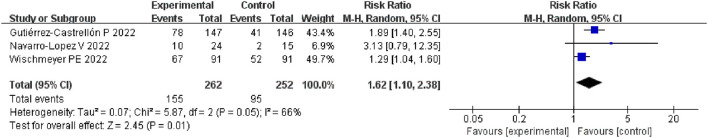
Forest plot of people with overall symptom improvement.

Three studies (23, 26, 27) reported the number of people with overall symptom improvement after treatment. The average improvement rate was 59.2% in the probiotics group and 37.7% in the non-probiotics group. Moderate heterogeneity was observed among included studies (degrees of freedom [df] = 2, *I*^2^ = 66%, *P* = 0.05). Accordingly, the random-effects model was selected for pooling effect sizes. The analysis revealed that probiotics had a better capacity to improve clinical symptoms than non-probiotics (RR = 1.62, 95% CI [1.10, 2.38], *P* = 0.01) (Figure 2).

### Meta-analysis of overall symptom duration

**Figure 3:**
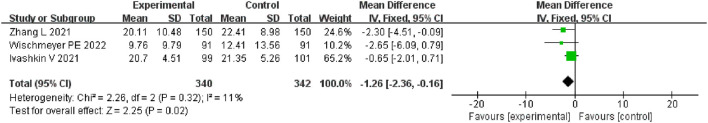
Forest plot of overall symptom duration.

Three studies (22, 25, 27) reported the duration (days) of overall symptoms. MD were calculated to analyze mean duration as it was continuous variable. Low heterogeneity was identified using Cochrane's Q test (df = 2, *I*^2^ = 11%, *P* = 0.32). The fixed-effects model was selected for pooling effect sizes. The results showed that probiotics shortened the duration of overall symptoms in patients with COVID-19, and it reached a statistical difference (MD = −1.26, 95% CI [−2.36, −0.16], *P* = 0.02) (Figure 3).

### Meta-analysis of C-reactive Protein

**Figure 4:**
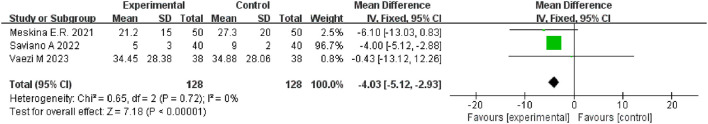
Forest plot of C-reactive protein.

Four studies (22, 28, 29, 31) reported the CRP serum level (mg/L). High heterogeneity was identified using Cochrane's Q test (df = 3, *I*^2^ = 91%, *P* < 0.00001). However, when we excluded one study (22), the heterogeneity between the studies was significantly reduced (df = 2, *I*^2^ = 0%, *P* = *0.72*). Through a fixed-effect analysis, the results showed that probiotics could reduce CRP serum level in patients with COVID-19 (MD = −4.03, 95% CI [−5.12, −2.93], *P* < 0.00001) (Figure 4).

### Meta-analysis of hospital stay

**Figure 5:**
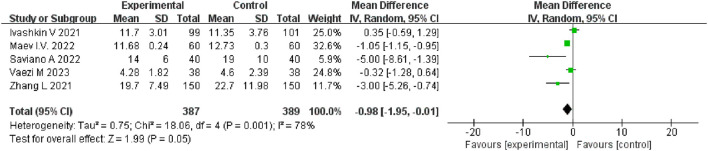
Forest plot of hospital stay.

Five studies (22, 25, 28, 30, 31) reported the hospital stay (days). A total of 776 patients included, 387 in the probiotics group and 389 in the non-probiotics group. High heterogeneity was observed among the five studies (df = 4, *I*^2^ = 78%, *P* = 0.001). Nevertheless, we selected random-effects model to pool the effect sizes. The results showed that probiotics reduced the average hospital stay of COVID-19 patients (MD = −0.98, 95% CI [-1.95, −0.01], *P* = *0.05*), and nearly reached statistical difference (Figure 5). In fact, the value of P was 0.047 through the auxiliary analysis of Stata 14.0, indicating that there was statistical difference between two groups. Heterogeneity decreased (df = 3, *I*^2^ = 69%, *P* = 0.02) when one study (22) was excluded, and the results (MD = −1.38, 95% CI [−2.44, −0.32], *P* = 0.01) were still stable.

### Subgroup analysis

We further analyzed the duration (days) of specific symptoms during the probiotic intervention (Table 3). Five studies (22–24, 29, 30) including 200 patients reported the duration of diarrhea. When we examined the two groups using Cochrane's Q test, no obvious heterogeneity was detected among the five studies. The duration of diarrhea in probiotics group was significantly shorter than that in non-probiotics group with fixed-effects (MD = −2.12, 95% CI [−2.41, −1.83], *P* < 0.00001). Similarly, we analyzed three studies (23, 29, 31) that reported respiratory symptoms of cough and shortness of breath. The results show that probiotics can improve cough (MD = −2.21, 95% CI [−4.56, 0.13], *P* = 0.06) and shortness of breath (MD = −1.37, 95% CI [−2.22, −0.53], P = 0.001), although there was no statistical difference in the initial analysis of cough. When we excluded a study (31) with the smallest sample size, the results of cough had statistical significance (MD = −3.66, 95% CI [−4.51, −2.81], *P* < 0.00001) and low heterogeneity (*I*^2^ = 44%). There was no significant difference in fever, headache, and weakness.

**Table 3:** Meta analysis results of specific symptoms.

	**df**	**Cochran's Q test**		**Statistical model**	**MD (days)**	**95% CI**	***P***
		**I** ^2^	**P**				
Diarrhea (21–23, 28, 29)	4	0%	0.45	Fixed-effect	−2.12	[−2.41,−1.83]	< 0.00001
Cough (22, 28, 30)	2	85%	0.001	Random-effects	−2.21	[−4.56,0.13]	0.06
Shortness of breath (22, 28, 30)	2	43%	0.17	Fixed-effect	−1.37	[−2.22,−0.53]	0.001
Fever (22, 24, 30)	2	91%	< 0.00001	Random-effects	−1.40	[−3.16,0.36]	0.12
Headache (22, 30)	1	97%	< 0.00001	Random-effects	−2.59	[−6.65,1.48]	0.21
Weakness (28, 30)	1	67%	0.08	Random-effects	−0.37	[−4.19,3.46]	0.85

MD, mean difference; df, degree of freedom; CI, confidence intervals.

### Publication bias test

As no outcome variable was included in all studies, both the diarrhea and hospital stay were selected to examine publication bias using Egger's test. The *P*-values for diarrhea and hospital stay were 0.913 and 0.972 (*P* > 0.05), respectively. They confirmed the stability of our results.

## Discussion

The binding of angiotensin converting enzyme 2 (ACE2) receptor to enter the human body has been reported as the pathogenesis of SARS-CoV-2 (32). ACE2 is abundant in epithelial cells of lung and intestine, indicating that there is a potential link between them (33). Although COVID-19 virus is mainly transmitted in respiratory tract and contact process, surviving virus particles have been also found in stool samples of infected patients (34). A study (35) involving 4,434 patients with COVID-19 showed that the pooled validity of gastrointestinal inventions was 11.51%. Positivity for COVID-19 in stool samples was observed in 41.50% of cases. The most frequent gastrointestinal symptom was diarrhea, followed by nausea/vomiting, poor appetite and abdominal pain. Moreover, it was reported that gastrointestinal symptom not only appeared in infected COVID-19 patients, but might even be earlier than typical respiratory symptoms (36, 37).

Probiotics have been gradually contemplated to improve the symptoms of COVID-19, even for patients with extremely severe illness (38). COVID-19 and intestinal microbiota can interact with each other (39). Some studies suggest that intestinal microbiota may be a predictor of COVID-19 severity (40, 41). Therefore, regulating intestinal flora seems to be an effective aspect in improving symptoms (42). In addition, probiotics may improve the immune level of COVID-19 patients. A RCT (43) on probiotic strain *Loigolobacillus coryniformis* K8 CECT 5711 showed that IgG levels in the *L. coryniformis* K8 group were significantly higher than placebo group in people over 60 years of age. For ages 85 and older, probiotic administration increased IgA antibody levels. Synodinou et al. (18) believed that probiotics acted by blocking the virus from invading and proliferating in host cells, by stimulating the immune response, and by suppressing the activation of NLRP3 inflammasome. Moreover, Anwar et al. (44) suggested that probiotic metabolites might have antiviral effects on COVID-19. Based on these aspects, probiotics seem to be beneficial for patients with COVID-19.

Through a bibliometric analysis, Xavier-Santos et al. (45) believed that probiotics, prebiotics, synbiotics, and postbiotics represented a promising adjuvant approach for improving the health of patients with COVID-19. A meta-analysis by Viana et al. (46) suggested that probiotic supplementation was effective in improving symptoms of COVID-19. However, outcome indicators contained few studies, most of which had only one or two articles. They claimed that there was a significant reduction in cough (RR = 0.56, 95% CI [0.37, 0.83]; *p* = 0.49; I^2^ = 0%), headache (RR = 0.17, 95% CI [0.05, 0.65]; *p* = 0.38; I^2^ = 0%), and diarrhea (RR = 0.33, 95% CI [0.12, 0.96]; *p* = 0.04; I^2^ = 76%) among COVID-19 patients using probiotics. Their research results seemed unconvincing. Therefore, a higher-quality evidence was still lacking, and we processed it.

Our study included inpatients and outpatients of COVID-19 from 7 countries. Among the 10 studies included, the maximum sample size is 300 and the minimum is 41. Our outcome indicators have richer and more reliable data than previous study. As the duration of specific symptoms in most of COVID-19 patients are short (< 10 days), most of studies have a long follow-up period (more than 10 days). The duration of symptom improvement seems to be more accurate than the number of people with symptom improvement. In addition, inflammatory indicators – CRP and hospital stay were included to analysis, which can further reflect the course of COVID-19 patients.

Through our meta-analysis, the results showed that probiotics could improve the overall symptoms of COVID-19 patients and shorten the duration of symptoms. For gastrointestinal symptoms, the pooled results of five studies (22–24, 29, 30) confirmed that probiotics could obviously reduce the duration of diarrhea in COVID-19 patients. Good consistency exists among the studies, indicating that the results are stable. For respiratory symptoms, probiotics improved cough and shortness of breath in COVID-19 patients. This might be related to the mechanism of probiotics supplementation on alleviating asthma symptoms via increasing Treg cells population. Probiotics supplementation could control T-helper 2-predominant and Th17 pro-inflammatory responses and improve respiratory function (11). Based on these results, we suggested that probiotics could improve the gastrointestinal symptoms and further improve the respiratory symptoms. This also confirms the theory of gut-lung axis. No obvious improvement was observed in other symptoms (including fever, headache, and weakness), although it was mentioned in Table 1 that probiotics might improve fatigue feelings by stimulating plasmacytoid dendritic cells and improve neurological disorders by stimulating neurite growth of SH-SY5Y through the gut-brain axis.

CRP may be a rapid, widely available, useful predictive factor for determining the severity of COVID-19 patients (47). Huang et al. (48) suggested that an elevated serum CRP were associated with a poor outcome in COVID-19. In a retrospective study conducted by Sadeghi-Haddad-Zavareh et al. (49), 429 patients diagnosed with COVID-19 was divided into severe (*n* = 175) and non-severe cases (*n* = 254). The results showed that the proportion of patients with increased CRP levels was significantly higher in severe cases than in non-severe patients, and patients with CRP >64.75 mg/L were more likely to have severe complications. Therefore, lowering the CRP serum level seems to improve the severity and progression of patients with COVID-19. Although in the four studies we included, both probiotics group and non-probiotics group had a lower CRP serum level than the baseline after treatment. The results of our meta-analysis showed that probiotics had a more significant ability to reduce CRP serum level than non-probiotics, acting as one way to reduce the inflammatory reaction of body. Of course, it was not ruled out that probiotics affected inflammatory parameters by reducing TGF-β1 concentrations, IL-8, increasing IL-5 and Il-10, and IFN-γ and IL-12 (50).

Most mild COVID-19 patients do not need hospitalization. However, for the elderly patients with serious basic diseases, hospitalization or transfer to ICU has become the best choice. A single center retrospective study (51) from Slovenia showed that the median length of stay on regular wards was 7.5 (IQR 5–13) days, and the median ICU length of stay was 6 (IQR 4–11) days. The probability of dying in 21 days was high as 14.4% (95% CI [10.9–18%]) at the regular ward and 43.6% at the ICU. In addition, an analysis (52) of risk factors and survival in patients with COVID-19 in northeastern Brazil showed that prolonged hospital length of stay was associated with a high risk of death. Therefore, it is important to help patients recover and discharge through effective treatment. Our pooled results from five studies showed that patients recovered faster and the hospital stay has been shortened through the treatment of probiotics.

However, some limitations exist in our study. First, we only included partial symptoms of gastrointestinal tract and respiratory tract, while some symptoms (such as vomiting, abdominal pain, myalgia) were abandoned due to the limitations of available data. The same is for other immunoinflammatory indicators (immunoglobulin, interleukin, procalcitonin). Second, intervention dosage and follow-up time are not completely consistent, and more studies with the same dosage and time cycle need to be included to maintain their consistency and minimize treatment bias. Some outcome indicators (including fever, headache, and weakness) have high heterogeneity and few included studies, which may lead to unstable results. Third, the lower limit of the absolute value of the confidence interval for the duration of overall symptoms and hospital stay are 0.16 and 0.01 using absolute value, respectively. According to the minimal clinically important difference (MCID) (53, 54), it becomes less likely that an appreciable numbers of patients will achieve important benefits in these aspects. The same applies to cough. Finally, the exact mechanisms of probiotics for COVID-19 patients need to be further investigated.

## Conclusion

Our findings suggest that probiotics could, to some extent, improve the overall symptoms, inflammatory reaction and shorten hospital stay of patients with COVID-19. Probiotics may improve gastrointestinal symptoms (such as improving the intestinal flora and reducing the duration of diarrhea) and further improve respiratory symptoms through the gut-lung axis. Probiotics are a kind of beneficial supplementations for patients with COVID-19.

## Data availability statement

The datasets presented in this study can be found in online repositories. The names of the repository/repositories and accession number(s) can be found in the article/ Supplementary material.

## Author contributions

Data curation and writing—review and editing: YT, HR, XW, and GF. Formal analysis: XW and RL. Funding acquisition: XW and TP. Investigation: YT and RL. Methodology: YT and HR. Project administration: YT, XW, and TP. Software: YT and XZ. Supervision and visualization: GF. Writing—original draft: YT, XW, and XZ. All authors contributed to the article and approved the submitted version.
