# Supplementary material for: Probiotics improve symptoms of patients with COVID-19 through gut-lung axis: a systematic review and meta-analysis
Source: Front Nutr. 2023 May 22;10:1179432. doi: 10.3389/fnut.2023.1179432 (PMC10239816; doi:10.3389/fnut.2023.1179432)
Supplement: Supplementary file 1 [file Data_Sheet_1.PDF]

Supplementary materials S1

Estimating the mean and standard deviation:

1. When a 95% CI is available for a sample in group of normal distribution or approximate normal distribution, range value of [μ-1.96σ, μ+1.96σ] is available. Therefore, we use it to calculate the standard deviation (SD). For example: [A =μ-1.96σ, B=μ+1.96σ].

$$\text{SD}=\sigma = \frac{B-A}{3.92}$$

(μ is the value of mean and σ expresses the SD).

2. When sample means and standard deviations for two different groups are available and the pooled mean and standard deviation are derived as follows:

$$\mu = \frac{n_A \times \mu_A + n_B \times \mu_B}{n_A + n_B}$$

$$\text{SD}=\sqrt{\frac{(n_A-1)\times SD_A^2+(n_B-1)\times SD_B^2}{n_A+n_B-2}}$$

(Where n_A and n_B are sample sizes of sample A and B, μ_A and μ_B are means of sample A and B, μ is the combined mean of A and B, while SD_A and SD_B are standard deviations of these two samples)

3. When the first quartile , the median, third quartile and sample size for interquartile range (IQR) are available, the pooled mean and standard deviation are derived as follows:

$$\text{SD} \approx \frac{q_3 - q_1}{2\emptyset^{-1}(\frac{0.75n - 0.125}{n + 0.25})}$$

$$\overline{X} \approx \left(0.7 + \frac{0.39}{n}\right)\frac{q_1 + q_3}{2} + \left(0.3 - \frac{0.39}{n}\right)m$$

(Where q_1 and q_3 are the first quartile and the third quartile, m is the median and n is the sample size. $\overline{X}$ is the estimated mean value)
